# DSD-Mamba: Dual-Stream Semantic Segmentation of Remote Sensing Imagery via Dense-Sparse Fusion

**DOI:** 10.3390/s26123864

**Published:** 2026-06-17

**Authors:** Xinyi Feng, Shaochen Jiang, Liejun Wang, Beibei Gao

**Affiliations:** School of Computer Science and Technology, Xinjiang University, Urumqi 830046, China; 107552503723@stu.xju.edu.cn (X.F.); 107552403898@stu.xju.edu.cn (B.G.)

**Keywords:** remote sensing image segmentation, high-resolution imagery, state space models, Mamba, Top-*k* selective aggregation, strip attention, dual-stream decoder, UAV imagery

## Abstract

High-resolution remote sensing image segmentation is important for urban mapping but remains challenging because of spectral ambiguity, large scale variations, fragmented elongated structures, and background interference. This study aims to improve semantic segmentation in complex aerial scenes by combining local feature extraction, selective multi-scale fusion, and global sequence modeling. We propose DSD-Mamba, an asymmetric dual-stream architecture with a ResNet-18 encoder. The Dense-Sparse Pyramid Fusion Module aligns multi-level features and applies dual Top-*k* selective value aggregation for cross-scale response filtering and background-response suppression. This Top-*k* operation is used as a feature-selection mechanism and is not intended to reduce the theoretical memory footprint of dense attention. Scale-Aware Strip Attention refines skip connections through horizontal and vertical dependency modeling, and the Dual-Stream Context Decoder combines a Mamba-based global branch with a CNN-based local branch during upsampling. Experiments were conducted on UAVid, ISPRS Vaihingen, and ISPRS Potsdam under a single-model inference protocol without test-time augmentation. DSD-Mamba achieved mIoU scores of 73.4%, 85.2%, and 87.2%, respectively. Ablation experiments on Vaihingen showed that DSPFM, SASA, and DSCD improved performance over the baseline when evaluated in this setting, with the full model reaching the highest mIoU. The method improves segmentation accuracy under the tested protocols, although its higher FLOPs indicate an accuracy-oriented rather than lightweight design.

## 1. Introduction

The increasing availability of high-resolution remote sensing imagery has advanced Earth observation applications, including urban planning, disaster monitoring, and automated mapping. In these applications, accurate pixel-level semantic segmentation is essential for interpreting complex urban scenes. However, parsing very-high-resolution remote sensing imagery remains challenging because objects vary substantially in scale, elongated structures are easily fragmented, and categories with similar spectral responses are often confused [1,2,3,4]. These challenges require models that can capture long-range contextual dependencies while preserving fine-grained local details.

Convolutional Neural Networks (CNNs) are effective at extracting local textures and structural cues, but their limited receptive fields make it difficult to model long-range dependencies in large aerial scenes [5,6]. Vision Transformers (ViTs) alleviate this limitation by explicitly modeling global interactions, yet dense self-attention introduces high computational cost, and patch-based tokenization may weaken boundary continuity for small or thin objects [7,8,9].

State Space Models (SSMs), especially Mamba [10], provide a promising alternative for efficient long-sequence modeling. Mamba uses a hardware-aware selective scan mechanism with linear complexity with respect to sequence length, and this concept has been adapted to vision tasks through visual state-space designs such as VMamba [11]. VM-UNet [12], Mamba-UNet [13], and Swin-UMamba [14] incorporated Mamba blocks into U-shaped architectures for dense prediction. In remote sensing, RS3Mamba [15] explored visual state-space modeling for large-scale spatial features, while UMFormer [16] investigated a lightweight hybrid CNN-Mamba framework for urban scene segmentation. Recent surveys further indicate that Mamba-based remote sensing models have rapidly developed toward scan-aware and hybrid CNN/Transformer/Mamba architectures, especially for high-resolution imagery and dense prediction tasks [17,18]. Recent segmentation-oriented studies have also explored this direction. CVMH-UNet integrates Vision Mamba with multi-scale and multi-frequency feature fusion for remote sensing image segmentation [19], while MAFMamba introduces multi-scale adaptive fusion and global–local Mamba modeling for high-resolution remote sensing semantic segmentation [20]. Although these recent studies have achieved promising progress, several limitations remain when they are applied to complex urban remote sensing scenes. First, global modeling modules, including Transformer attention and visual state-space scanning, can capture long-range dependencies but may still aggregate redundant responses from large homogeneous background regions, introducing noisy contextual information. Second, elongated man-made structures, such as roads, rivers, railways, and bridges, require explicit directional context modeling; however, many existing models rely mainly on isotropic convolution, window attention, or general sequence scanning, which may not sufficiently preserve axial continuity. Third, standard decoder designs usually fuse global semantic features and local spatial details through direct concatenation, addition, or convolution, without explicitly decoupling global semantic consistency from local boundary refinement. These limitations motivate a more targeted architecture for remote sensing segmentation. To address these gaps, we propose DSD-Mamba, an asymmetric dual-stream framework with dense-sparse feature fusion for remote sensing semantic segmentation. At the encoder–decoder bottleneck, the Dense-Sparse Pyramid Fusion Module (DSPFM) performs multi-scale feature alignment and Top-*k* selective value aggregation for cross-scale context modeling. This operation is designed to suppress redundant background responses during feature aggregation rather than to reduce the theoretical memory footprint of dense self-attention. In the skip connections, Scale-Aware Strip Attention (SASA) introduces horizontal and vertical contextual cues to enhance the continuity of elongated structures. During upsampling, the Dual-Stream Context Decoder (DSCD) combines a Mamba-based global semantic branch with a CNN-based local-detail branch, allowing the decoder to jointly use long-range context and fine-grained boundary information.

To avoid ambiguity, the term “dense-sparse” in this work describes the feature fusion behavior rather than an asymptotically sparse computational architecture. Specifically, the dense part refers to multi-scale feature alignment, residual CNN aggregation, and triangular cross-scale interactions among encoder features, whereas the sparse part refers to Top-*k* selective response aggregation used to reduce redundant background responses during feature aggregation.

The main contributions of this work are summarized as follows:

- We propose DSD-Mamba, a hybrid U-shaped framework for high-resolution remote sensing semantic segmentation. The model integrates CNN-based local representation, Mamba-based global sequence modeling, and Top-*k* selective feature aggregation to address complex urban scenes.
- We design the Dense-Sparse Pyramid Fusion Module (DSPFM) for bottleneck feature fusion. DSPFM performs dense multi-scale alignment and triangular cross-scale interaction, followed by Top-*k* selective value aggregation to reduce the influence of redundant background responses. We clarify that this operation is used for feature selection rather than theoretical memory reduction.
- We introduce Scale-Aware Strip Attention (SASA) in the skip-fusion pathway. SASA models horizontal and vertical contextual dependencies and is designed to improve the geometric continuity of elongated regions such as roads, rivers, and bridges.
- We develop the Dual-Stream Context Decoder (DSCD), which combines a Mamba-based global semantic branch with a CNN-based local-detail branch. This design decouples global semantic consistency from local boundary refinement during upsampling.
- We conduct experiments on UAVid, ISPRS Vaihingen, and ISPRS Potsdam, together with ablation and complexity analyses, to evaluate the accuracy–cost trade-off of the proposed method under the adopted high-resolution evaluation protocols.

## 2. Related Work

### 2.1. Machine-Learning-Based Land-Use Identification

Machine-learning-based land-use and land-cover identification has long been an important topic in remote sensing and geospatial analysis. Earlier studies commonly relied on integrated data sources, spectral and spatial descriptors, and supervised learning models to infer land-use categories from satellite imagery and auxiliary geographic information. For example, Meedeniya et al. [21] presented an automated land-use classification methodology based on integrated data and learning models, highlighting the importance of combining remote sensing observations with additional contextual information for land-use and land-cover identification. Related studies also explored the integration of satellite imagery with geographic or social sensing data, such as Foursquare-based information, to enhance land-use information generation.

These ML-based studies provide an important foundation for remote sensing scene understanding because they emphasize data integration, discriminative feature representation, and supervised classification. However, many land-use identification approaches are designed for image-level, region-level, or object-level classification, where the goal is to assign a land-use label to a spatial unit. In contrast, high-resolution semantic segmentation requires pixel-level prediction and therefore demands more precise boundary recovery, stronger multi-scale context modeling, and better preservation of thin or elongated structures. This distinction motivates the development of deep dense-prediction architectures that can jointly model global context and local spatial details.

### 2.2. CNN-Based Semantic Segmentation

Early semantic segmentation models were primarily built on Convolutional Neural Networks (CNNs). Fully convolutional networks and U-Net-style encoder–decoder architectures established the basic paradigm for dense prediction by combining high-level semantic features with low-level spatial details [6,22,23]. In remote sensing, where object scales vary substantially, methods such as SwiftNet [24] and MANet [25] introduced multi-scale aggregation strategies to handle scale variations. ABCNet [3] enhanced boundary definition through bilateral context attention, while edge-aware networks such as EaNet [26] and ResUNet-a [27] focused on refining object contours. Despite their efficiency in extracting local textures, CNN-based models remain limited in modeling long-range dependencies over large aerial scenes [5].

### 2.3. Transformer-Based Dense Prediction

To overcome the locality constraints of CNNs, Vision Transformers (ViTs) [7] were introduced to model global interactions through self-attention. TransUNet [8] adopted a hybrid design by combining CNN encoders with Transformer bottlenecks, thereby integrating local inductive bias and global context. Hierarchical architectures such as Swin Transformer [28] and SegFormer [29] further used shifted windows and sequence reduction to reduce the quadratic complexity ($O(N^{2})$) of dense attention. In remote sensing, UNetFormer [4] and Swin-UperNet [28] have shown strong performance by leveraging global modeling capabilities. Other methods, including BANet [30] and CMTFNet [31], explored bilateral awareness and multi-scale fusion. However, the computational cost of attention remains a bottleneck for high-resolution aerial imagery, and patch-based tokenization can weaken fine-grained structural continuity [9].

### 2.4. State Space Models for Vision

Structured State Space Models (SSMs), particularly Mamba [10], have recently emerged as an efficient alternative to Transformers for long-sequence modeling. Mamba uses a hardware-aware selective scan mechanism with linear complexity with respect to sequence length, and this idea has been adapted to vision tasks through visual state-space designs such as VMamba [11]. VM-UNet [12], Mamba-UNet [13], and Swin-UMamba [14] incorporated Mamba blocks into U-shaped architectures for dense prediction. In remote sensing, RS3Mamba [15] explored visual state-space modeling for large-scale spatial features, while UMFormer [16] investigated a lightweight hybrid CNN-Mamba framework for urban scene segmentation. Recent studies further extended Mamba-based remote sensing segmentation. CVMH-UNet combines Vision Mamba with multi-scale and multi-frequency feature fusion [19], while MAFMamba introduces multi-scale adaptive fusion and global–local Mamba modeling for high-resolution remote sensing segmentation [20]. Recent surveys also summarize the rapid development of Vision Mamba in remote sensing and emphasize the importance of scan-aware hybrid designs [17,18]. Although these methods are promising, complex urban remote sensing scenes still present challenges. Global scanning may accumulate redundant background responses, elongated man-made structures require directional geometric modeling, and standard decoding strategies may not sufficiently separate global semantic consistency from local boundary refinement. These observations motivate DSPFM for selective context aggregation, SASA for axial skip-feature processing inspired by strip pooling [32], and DSCD for dual-stream decoding.

## 3. Methodology

### 3.1. Overall Architecture

DSD-Mamba is designed as a U-shaped semantic segmentation framework that combines CNN-based local feature extraction, Top-*k* selective cross-scale aggregation, and Mamba-based global sequence modeling.Given an input image $I\in R^{3\times H\times W}$, a ResNet-18 encoder [5] extracts four hierarchical feature maps,

(1) $$\left\{E_{1},E_{2},E_{3},E_{4}\right\}=E\left(I\right),$$

where $E_{1}$, $E_{2}$, $E_{3}$, and $E_{4}$ correspond to progressively lower spatial resolutions and stronger semantic abstraction. For a 1024×1024 input patch, these features are produced at approximately 1/4, 1/8, 1/16, and 1/32 of the input resolution with channel dimensions of 64, 128, 256, and 512, respectively.

**Figure 1:**
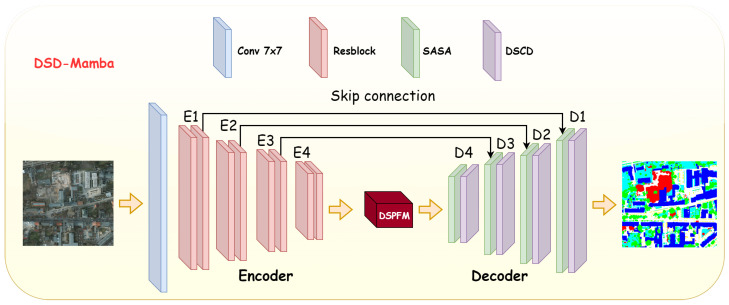
The overall architecture of DSD-Mamba. The encoder provides multi-level features; DSPFM performs bottleneck cross-scale fusion; SASA processes skip features with axial attention; and DSCD combines Mamba-based global context with CNN-based local detail.

As shown in Figure 1, the network contains three task-oriented components. First, the Dense-Sparse Pyramid Fusion Module (DSPFM) is placed at the bottleneck to align encoder features from different scales and construct cross-scale interactions using the proposed Multi-Scale Selective Cross-Attention (MSC) unit. Second, the Scale-Aware Strip Attention (SASA) module is used in decoder skip fusion to introduce horizontal and vertical context before channel projection. Third, the Dual-Stream Context Decoder (DSCD) reconstructs high-resolution predictions by combining a Mamba-based global branch with a CNN-based local branch. This design follows the reconstruction principle of U-Net, but uses Top-*k* selective cross-scale interaction and global–local context fusion instead of plain skip concatenation and convolutional decoding. The final segmentation logits are generated by a 1×1 convolution.

### 3.2. Dense-Sparse Pyramid Fusion Module (DSPFM)

High-resolution remote sensing scenes contain objects with large scale variation, such as buildings, roads, vehicles, trees, and impervious surfaces. Directly using only the deepest encoder feature may lose fine structures, whereas naively concatenating all encoder features can introduce redundant low-level responses. For this reason, DSPFM performs scale alignment, residual CNN fusion, and  Top-*k* selective cross-scale aggregation in a unified bottleneck module.

#### 3.2.1. Scale Alignment and CNN Residual Fusion

Let $C^{\prime }=128$ denote the bottleneck channel dimension. In DSPFM, the  four encoder features are aligned to the spatial resolution of the deepest encoder feature $E_{4}$ and projected to the same channel dimension $C^{\prime }$. This alignment scale is chosen because DSPFM is designed as a bottleneck-level context aggregation module rather than a high-resolution boundary-recovery module. The deepest feature $E_{4}$ contains the strongest semantic abstraction, and using its spatial resolution enables compact cross-scale interaction for semantic context modeling and redundant-response suppression.

This design also considers computational feasibility. Although Top-*k* masking is used during value aggregation, the current MSC implementation still computes the dense affinity logits before masking. Therefore, the memory and computation of the interaction stage are closely related to the number of spatial tokens. Let the alignment resolution be $H_{b}\times W_{b}$ and $N_{b}=H_{b}W_{b}$. The affinity matrix scales approximately with $O(N_{b}^{2})$. For a 1024×1024 input patch, alignment at the 1/32 scale produces 32×32=1024 spatial tokens. If the alignment were performed at the 1/16, 1/8, or 1/4 scale, the number of tokens would increase to 4096, 16,384, or 65,536, and the corresponding affinity matrix would become approximately 16×, 256×, or 4096× larger, respectively. Thus, aligning to the $E_{4}$ resolution provides a practical trade-off between cross-scale semantic aggregation and computational cost.

The potential loss of high-resolution spatial details is mitigated by the overall encoder–decoder design. Shallow encoder features are still delivered to the decoder through SASA-enhanced skip connections, and the DSCD local branch further refines local boundaries and high-frequency details during upsampling. Therefore, DSPFM focuses on compact multi-scale semantic aggregation at the bottleneck, while fine spatial recovery is mainly handled by the skip-fusion and decoder stages. This design choice is further examined in the alignment-resolution ablation study in Section 4.5, where the default 1/32 alignment is compared with an intermediate 1/16 alignment.

Specifically, the shallow features are downsampled by depthwise separable convolution blocks with stride 2, while the deepest feature is projected by a 1×1 convolution:

(2) $$\begin{array}{cc} P_{1} & \;=D_{2}\left(D_{2}\left(D_{2}\left(E_{1}\right)\right)\right),\\ P_{2} & \;=D_{2}\left(D_{2}\left(E_{2}\right)\right),\\ P_{3} & \;=D_{2}\left(E_{3}\right),\\ P_{4} & \;=C_{1\times 1}\left(E_{4}\right), \end{array}$$

where $D_{2}\left(\cdot \right)$ denotes a stride-2 depthwise separable convolution followed by batch normalization and SiLU activation, and $C_{1\times 1}\left(\cdot \right)$ denotes a 1×1 convolution with batch normalization and activation. The aligned features $\left\{P_{1},P_{2},P_{3},P_{4}\right\}$ have the same shape $R^{C^{\prime }\times H/32\times W/32}$, which enables the subsequent MSC units to compute cross-scale interactions on a compact bottleneck token grid.

A convolutional residual branch is used to preserve local spatial continuity after scale alignment:

(3) $$F_{cnn}=R\left(C_{1\times 1}\left([P_{4},P_{3},P_{2},P_{1}]\right)\right),$$

where [·] denotes channel-wise concatenation and R(·) consists of two residual identity blocks. This CNN branch provides a local representation that complements the Top-*k* selective interaction branch after downsampling.

#### 3.2.2. MSC-Based Selective Dense-Sparse Cross-Scale Interaction

The selective interaction branch models pairwise relations among all aligned pyramid features. Following the implementation order, we define

(4) $$S_{1}=P_{4},\;S_{2}=P_{3},\;S_{3}=P_{2},\;S_{4}=P_{1}.$$

For each pair $\left(S_{i},S_{j}\right)$ with i≤j, an MSC unit computes a Top-*k* filtered cross-attention feature $Z_{ij}=\mathrm{MSC}\left(S_{i},S_{j}\right)$. The triangular pairing strategy produces ten cross-scale interaction maps, including self-scale and cross-scale relations:

(5) $$Z=\{Z_{ij}|1\leq i\leq j\leq 4\}.$$

The output of the selective branch is obtained by concatenating these ten maps and reducing the channels by a 1×1 convolution:

(6) $$F_{msc}=C_{1\times 1}\left([Z_{11},Z_{12},\ldots ,Z_{44}]\right).$$

Finally, DSPFM combines the MSC interaction branch and the CNN residual branch:

(7) $$F_{b}=F_{msc}+F_{cnn},$$

where $F_{b}$ is the bottleneck feature delivered to the decoder. The term “dense-sparse” is used to describe the feature fusion process rather than the theoretical computational complexity. Specifically, “dense” refers to the multi-scale feature alignment, CNN residual fusion, and triangular cross-scale interaction among the aligned pyramid features, while “sparse” refers to the Top-*k* selective value aggregation inside each MSC unit. It should be noted that the current implementation still computes the dense affinity logits $QK^{T}$ before Top-*k* masking. Therefore, DSPFM is primarily a selective aggregation mechanism for suppressing redundant responses, rather than a low-complexity attention approximation or a memory-efficient replacement for dense self-attention.The use of the $E_{4}$ resolution should also be understood as a practical bottleneck-level design choice, which improves the feasibility of cross-scale interaction while delegating high-resolution spatial detail recovery to SASA-guided skip fusion and DSCD-based decoding. For clarity, the MSC unit is described as follows. Given a query feature $X\in R^{B\times C^{\prime }\times H_{b}\times W_{b}}$ and a context feature $Y\in R^{B\times C^{\prime }\times H_{b}\times W_{b}}$, MSC first extracts multi-scale context from *Y* using average pooling with kernel sizes {3,5,7}:

(8) $$\tilde{Y}=\mathrm{LN}\left(\mathrm{Flatten}\left(\sum_{r\in \{3,5,7\}}{\mathrm{AvgPool}}_{r\times r}\left(Y\right)\right)\right).$$

The query tokens are obtained from *X*, and the key/value tokens are obtained from $\tilde{Y}$:

(9) $$Q=X_{f}W_{q},\;\left[K,V\right]=\tilde{Y}W_{kv},$$

where $X_{f}=\mathrm{Flatten}\left(X\right)$. The attention logits are computed as

(10) $$L=\frac{QK^{T}}{\sqrt{d}},$$

where *d* is the head dimension. Instead of aggregating values from all positions, MSC uses two Top-*k* branches with different sparsity ratios. For branch m∈{1,2}, the number of selected context tokens is $\left\lfloor N_{y}/r_{m}\right\rfloor$, where $r_{1}=2$ and $r_{2}=3$ in the implementation. The Top-*k* masked attention used for selective value aggregation is therefore

(11) $$A_{m}=\mathrm{Softmax}\left(\mathrm{MaskTopK}\left(L,\left\lfloor \frac{N_{y}}{r_{m}}\right\rfloor \right)\right),$$

where non-selected logits are set to −∞ before the softmax operation. The two branches are combined using learnable scalar weights $\gamma_{1}$ and $\gamma_{2}$:

(12) $$\mathrm{MSC}\left(X,Y\right)=\mathrm{Proj}\left(\gamma_{1}A_{1}V+\gamma_{2}A_{2}V\right).$$

**Figure 2:**
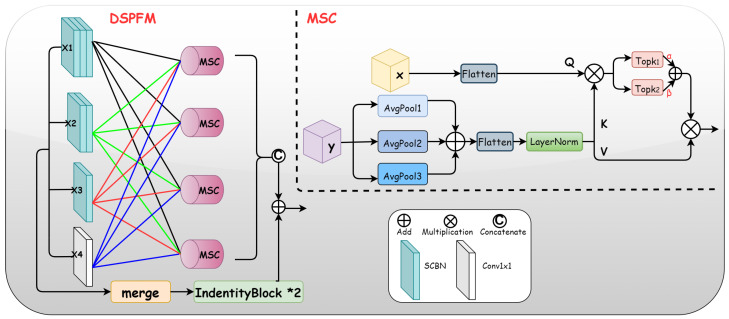
DSPFM consists of a CNN residual branch and an MSC-based FL FL Top-*k* selective interaction branch. The MSC unit uses multi-scale pooled context features and dual Top-*k* FL FL selective aggregation for cross-scale response FL FL filtering. The Top-*k* operation is used for response selection rather than reducing the theoretical memory footprint of dense attention.

The first branch retains a broader set of context tokens, while the second branch selects a more compact subset of high-response tokens. This design is intended to reduce the influence of redundant responses during value aggregation. Thus, the sparse operation in MSC should be understood as response selection during value aggregation, not as a claim of reduced asymptotic attention complexity or reduced memory footprint (see Figure 2).

### 3.3. Scale-Aware Strip Attention (SASA)

Skip features in U-shaped segmentation networks carry important boundary and texture information, but they may also contain semantic noise and local discontinuities. This problem is particularly evident in remote sensing imagery, where roads, bridges, rivers, and roof edges often appear as elongated structures. SASA is introduced to provide directional context in the skip-fusion pathway before channel projection.

Given an input feature $X\in R^{B\times C\times H\times W}$, SASA first applies a 1×1 convolution to obtain a channel-aligned feature $X_{0}$. Then three convolutional kernels with different receptive fields are used to capture scale-aware local context:

(13) $$X_{s}=C_{3\times 3}\left(X_{0}\right)+C_{5\times 5}\left(X_{0}\right)+C_{7\times 7}\left(X_{0}\right).$$

Compared with a single fixed kernel, this multi-kernel design offers receptive fields of different sizes for skip features.

SASA then computes strip attention along the two spatial axes. For one axis, the feature is permuted so that responses are aggregated along the orthogonal spatial dimension. Max pooling and average pooling are used jointly to summarize salient and contextual responses:

(14) $$D_{h}=\left[{\mathrm{Max}}_{w}\left(X_{s}\right),{\mathrm{Avg}}_{w}\left(X_{s}\right)\right],$$

where $D_{h}$ is a two-statistic descriptor for the horizontal-axis attention. A 1×1 convolution reduces the descriptor, followed by a channel-wise 1D convolution, batch normalization, and sigmoid activation:

(15) $$A_{h}=\sigma \left(\mathrm{BN}\left(\mathrm{DWConv1D}\left(C_{1\times 1}\left(D_{h}\right)\right)\right)\right).$$

**Figure 3:**
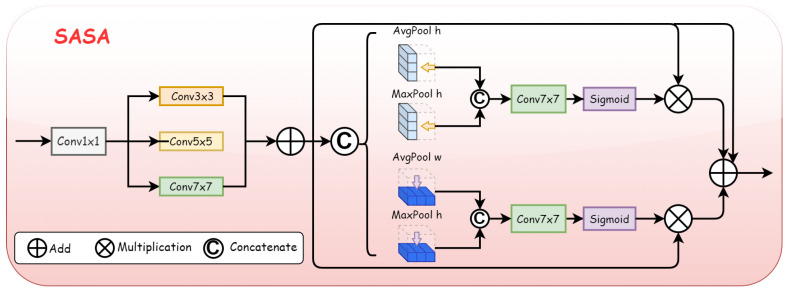
SASA processes skip-fusion features with multi-kernel local context and horizontal/vertical strip attention. The module provides axial context while keeping the computation localized.

The same operation is applied along the vertical axis to obtain $A_{v}$. The final SASA output is

(16) $$Y_{sasa}=X_{s}+X_{s}⊙A_{h}+X_{s}⊙A_{v},$$

where ⊙ denotes broadcast multiplication. In the decoder, when an upsampled feature is concatenated with an encoder skip feature, SASA processes the concatenated tensor and a subsequent 1×1 convolution projects it to the decoder channel dimension. Thus, SASA acts as a directional refinement unit rather than a separate semantic segmentation head (see Figure 3).

### 3.4. Dual-Stream Context Decoder (DSCD)

The decoder must recover high-resolution predictions while retaining semantic consistency. Pure convolutional decoding is effective for edges and local textures but has limited ability to propagate long-range context. In contrast, sequence modeling can capture broader dependencies but may be less sensitive to fine boundary details. DSCD is designed around two complementary streams at each decoder stage: a Mamba-based global stream and a CNN-based local stream.

Let $X_{t}$ denote the input of the *t*-th decoder stage. For the first stage, $X_{t}$ is the bottleneck feature $F_{b}$. For later stages, the upsampled decoder feature is concatenated with the corresponding encoder skip feature, refined by SASA, and projected to the target decoder dimension. Each DSCD stage then computes

(17) $$F_{g}=G\left(X_{t}\right),\;F_{l}=L\left(X_{t}\right),$$

where G(·) and L(·) denote the global Mamba stream and the local CNN stream, respectively.

#### 3.4.1. Global Mamba Stream

The global stream models long-range dependencies using a Mamba block. To keep the state-space operation compact and stable, the input channels are split into four equal groups. This channel grouping reduces the feature dimension processed by each state-space operation while preserving the spatial size of the decoder feature map:

(18) $$X_{t}=\left[X_{t}^{\left(1\right)},X_{t}^{\left(2\right)},X_{t}^{\left(3\right)},X_{t}^{\left(4\right)}\right].$$

Each group is flattened from a 2D feature map into a 1D spatial token sequence and then processed by the same one-layer Mamba mixer:

(19) $$G^{\left(q\right)}={\mathrm{Reshape}}^{-1}\left(\mathrm{Mamba}*\theta \left(\mathrm{Flatten}(X_{t}^{\left(q\right)})\right)\right),\;q=1,2,3,4,$$

where Mamba∗θ denotes the shared Mamba mixer with the same parameter set θ for all channel groups.

Here, the same Mamba mixer is shared among the four channel groups. This design is adopted for three reasons. First, the four groups are channel partitions of the same decoder feature $X_{t}$ and share the same spatial layout; they are not treated as semantically independent branches. A shared mixer therefore encourages consistent spatial transition modeling across channel groups. Second, using independent Mamba mixers would introduce four separate sets of state-space parameters at each decoder stage, increasing the parameter count and the risk of overfitting, especially on remote sensing datasets with limited original scenes and strong spatial correlation among cropped patches. Third, the purpose of channel grouping is to keep the state-space operation compact rather than to create separate expert branches. The group outputs are concatenated after Mamba processing and then fused with the CNN-based local branch, allowing complementary channel information to be integrated in the subsequent global–local fusion. Therefore, the shared Mamba mixer is used as a parameter-efficient and stable design choice. We do not claim that it is universally superior to independent group-specific mixers, which may provide higher capacity and will be investigated in future work.

The four outputs are concatenated to form the global representation:

(20) $$F_{g}=\left[G^{\left(1\right)},G^{\left(2\right)},G^{\left(3\right)},G^{\left(4\right)}\right].$$

In the Mamba block, the spatial feature map is serialized into $H_{t}W_{t}$ tokens, normalized by LayerNorm, processed by the selective state-space mixer, and reshaped back to the original 2D layout. The channel-group design avoids applying one large state-space block over the full channel dimension and allows different channel subsets to model complementary global dependencies.

#### 3.4.2. Local Context Stream

The local stream is implemented by the LocalFeature block. It first combines a 1×1 convolution and a depthwise separable 3×3 convolution to obtain a locally enhanced feature:

(21) $$X_{l}^{0}=C_{1\times 1}\left(X_{t}\right)+{\mathrm{SepConv}}_{3\times 3}\left(X_{t}\right).$$

Then three residual identity blocks with dilation rates 1, 2, and 3 are applied in a cascaded manner:

(22) $$X_{l}^{1}=R_{d=1}\left(X_{l}^{0}\right),\;X_{l}^{2}=R_{d=2}\left(X_{l}^{1}\right),\;X_{l}^{3}=R_{d=3}\left(X_{l}^{2}\right).$$

The three dilation levels are concatenated and projected by a 1×1 convolution:

(23) $${\bar{F}}_{l}=C_{1\times 1}\left([X_{l}^{1},X_{l}^{2},X_{l}^{3}]\right).$$

A channel attention followed by a spatial attention map is used to emphasize informative local responses, and the result is added back to ${\bar{F}}_{l}$. Consistent with the implementation, channel attention first reweights ${\bar{F}}_{l}$, spatial attention is then computed on the channel-reweighted feature, and the resulting attentive feature modulates ${\bar{F}}_{l}$ in a residual form:

(24) $$U_{c}=A_{c}\left({\bar{F}}_{l}\right)⊙{\bar{F}}_{l},$$

(25) $$U_{s}=A_{s}\left(U_{c}\right)⊙U_{c},$$

(26) $$F_{l}={\bar{F}}_{l}+{\bar{F}}_{l}⊙U_{s},$$

where $A_{c}$ and $A_{s}$ denote channel and spatial attention, respectively. This branch provides short-range texture and edge-related cues for decoder reconstruction.

#### 3.4.3. Cosine-Guided Global–Local Fusion

After obtaining the global feature $F_{g}$ and the local feature $F_{l}$, DSCD fuses them through a cosine-guided residual embedding operation. The  purpose of this operation is not to suppress regions where local textures and global contexts disagree. In high-resolution remote sensing imagery, such disagreement may appear around object boundaries, small targets, shadows, or semantic transition regions. Therefore, the cosine-guided map is used as a soft consistency modulation weight for the refinement branch, rather than as a hard suppression mask.

The two features are first summed and smoothed by a depthwise separable convolution:

(27) $$F_{s}={\mathrm{SepConv}}_{3\times 3}\left(F_{g}+F_{l}\right).$$

A soft consistency map is then computed by channel-wise cosine similarity:

(28) $$S\left(h,w\right)=\sigma \left(\frac{F_{g}{\left(:,h,w\right)}^{T}F_{l}\left(:,h,w\right)}{|F_{g}{\left(:,h,w\right)|}_{2}{\left|F_{l}\left(:,h,w\right)\right|}_{2}+\epsilon }\right),$$

where $S\in R^{B\times 1\times H_{t}\times W_{t}}$ is broadcast along the channel dimension, and σ(·) denotes the sigmoid function. Since *S* is obtained from a sigmoid-activated cosine similarity, it provides a soft modulation weight instead of a binary decision.

The fused feature is obtained as

(29) $$F_{e}={\mathrm{SepConv}}_{3\times 3}\left(F_{s}⊙S\right)+F_{s}.$$

In this formulation, the term $F_{s}⊙S$ performs soft consistency-guided modulation. Regions where global semantic cues and local texture cues are consistent receive stronger refinement, while regions with disagreement are only down-weighted in the modulation branch to avoid over-amplifying unreliable responses. Importantly, these regions are not removed from the representation because the residual term $+F_{s}$ preserves the original fused global–local information.

The output of the decoder stage uses an additional global residual connection and is then upsampled by a factor of 2:

(30) $$X_{t+1}={\mathrm{Up}}_{2\times }\left(F_{e}+F_{g}\right).$$

**Figure 4:**
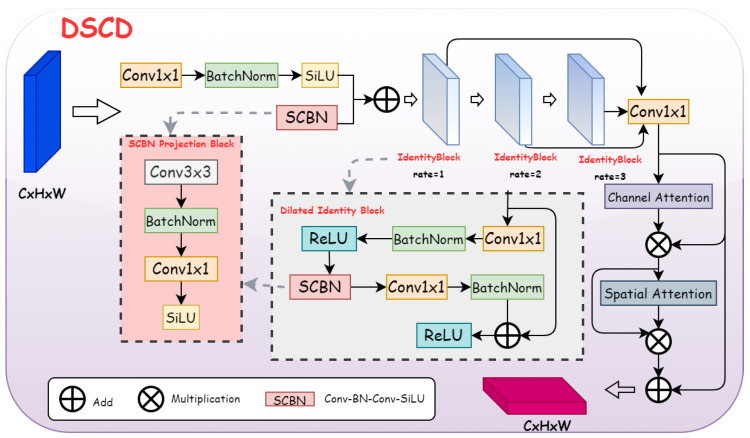
DSCD uses a Mamba-based global stream, a dilated CNN local stream, and cosine-guided feature embedding. The fused feature is residually connected with the global stream and progressively upsampled.

The additional residual propagation of $F_{g}$ further preserves global semantic context. Therefore, the cosine-guided fusion should be interpreted as confidence-weighted residual refinement rather than direct suppression of local–global disagreement. After four decoder stages, a final 1×1 convolution maps the feature to the number of semantic classes (see Figure 4).

## 4. Experiments

### 4.1. Datasets and Data Processing

To evaluate DSD-Mamba under different high-resolution remote sensing scenarios, experiments were conducted on three public benchmarks with a unified data processing pipeline. Given the large spatial size of the original scenes, a patch-based strategy was adopted for both training and inference.

UAVid Dataset [1]: This dataset features 300 high-resolution (3840×2160) densely annotated images, systematically sampled from 30 complex oblique UAV video sequences encompassing 8 semantic classes. Following standard evaluation protocols, 150 images (from 15 sequences) were used for training, and 50 images (from 5 sequences) were utilized for validation. As the ground truth of the official test set is withheld for server benchmarking, all quantitative comparisons in this study are reported on the official validation set. During pre-processing, the original images were first zero-padded in width to 4096×2160. Subsequently, a systematic hard-cropping strategy extracted the bottom-right 4096×2048 region so that both spatial dimensions were divisible by 1024, and the region was then partitioned into 1024×1024 patches without overlap. The padded background regions in the masks were assigned an Ignore Label (value 255) to prevent invalid gradient back-propagation.

ISPRS Potsdam Dataset [2]: This benchmark consists of 38 True Orthophotos (6000×6000). The widely adopted standard academic split was followed: 24 orthophotos were allocated for training, and the remaining 14 orthophotos were reserved for testing. To handle the non-divisible spatial resolution, an adaptive dynamic padding strategy was employed, zero-padding the bottom-right boundaries to 6144×6144. The enlarged images were subsequently cropped into 1024×1024 patches.

ISPRS Vaihingen Dataset [2]: This benchmark contains 33 Infrared-Red-Green (IRRG) tiles. Following the adopted benchmark split, the 16 publicly labeled tiles with IDs 1, 3, 5, 7, 11, 13, 15, 17, 21, 23, 26, 28, 30, 32, 34, and 37 were used for training, and the other 17 tiles were used as the test split in this study. Only the IRRG orthophotos were used; DSM data were not included. The tiles were dynamically padded using the aforementioned strategy and cropped into 1024×1024 patches without overlap.

### 4.2. Implementation Details and Inference Strategy

All experiments were implemented in PyTorch and trained on a single NVIDIA RTX 3090 GPU. The ResNet-18 backbone was initialized with SWSL-pretrained weights [33], while the newly introduced convolutional modules were initialized using Kaiming normal initialization [34]. The Mamba blocks used their default linear and normalization initialization.

For a fair comparison, the baselines that were re-implemented in our experiments followed a consistent initialization and pretraining policy. Specifically, when a model used a standard visual backbone, the corresponding publicly available pretrained weights were adopted when available and applicable, following the same controlled protocol used for DSD-Mamba. Newly introduced convolutional layers, decoder layers, and segmentation heads were initialized using Kaiming normal initialization. Transformer- and Mamba-related layers followed their official default initialization schemes. No additional remote-sensing test-set pretraining, medical-domain pretrained weights, or task-specific pretrained Mamba weights were used for any re-implemented baseline. This setting reduces the influence of inconsistent initialization or task-specific pretraining on the comparison.

Spectral-Spatial Input Adaptation: The backbone keeps the original first convolutional layer with three input channels. UAVid frames were used as RGB inputs. For both ISPRS Vaihingen and ISPRS Potsdam, the IRRG bands were mapped to the three input channels in the order Infrared, Red, and Green. No DSM information or four-channel RGBIR input was used. The network was trained end-to-end to adapt the three-channel filters to the remote sensing domain.

Optimizer and Learning Rate Schedule: A differential learning rate strategy was employed for all datasets. The  base learning rate was set to $6\times 10^{-4}$ for the decoder components, while a reduced learning rate of $6\times 10^{-5}$ was applied to the pre-trained ResNet-18 backbone to avoid disrupting the transferred low-level representations. AdamW [35] was used with a weight decay of 0.01, and the optimizer was wrapped with Lookahead [36] to improve training stability. The learning rate scheduler was selected according to the dataset scale, spatial correlation of cropped patches, and total training length. For UAVid, a standard cosine annealing schedule was adopted because the dataset contains diverse high-resolution UAV video-frame scenes and showed stable convergence with a monotonic learning rate decay over 400 epochs. For ISPRS Vaihingen and ISPRS Potsdam, cosine annealing with warm restarts was used because these tile-based aerial datasets contain fewer original scenes and stronger spatial correlation among cropped patches. Periodic warm restarts can re-increase the learning rate after convergence plateaus and help stabilize optimization during long training, especially for the 600-epoch Potsdam setting. These scheduler choices were adopted to obtain stable convergence under the adopted protocols and are not claimed to be globally optimal hyperparameter settings.

The training regimens and loss functions were set as follows:

- UAVid Dataset: The network was trained for 400 epochs with a batch size of 4. The loss combined Soft Cross-Entropy with a label smoothing factor of 0.05 and Dice Loss [37] with equal weights. A cosine annealing learning rate scheduler was used with $T_{\max}=400$ [38].
- ISPRS Vaihingen Dataset: The model was trained for 250 epochs with a batch size of 4. The loss was the same joint Soft Cross-Entropy and Dice Loss. Cosine annealing with warm restarts was used with $T_{0}=15$ and $T_{\mathrm{mult}}=2$ [38].
- ISPRS Potsdam Dataset: The model was trained for 600 epochs with a batch size of 8. Cosine annealing with warm restarts was used with $T_{0}=15$ and $T_{\mathrm{mult}}=2$. Dice Loss with a smoothing factor of 0.05 was used to optimize regional overlap.

During testing, all three datasets were evaluated using the same single-model inference protocol without test-time augmentation (TTA). Each 1024×1024 test patch was predicted once, and the final label map was obtained by pixel-wise argmax. No rotation, flipping, multi-scale inference, or $D_{4}$ test-time ensemble was used for UAVid, ISPRS Vaihingen, or ISPRS Potsdam. Predicted patches were stitched back to reconstruct the final scene-level prediction. Padded pixels and ignore labels were excluded from metric computation.

### 4.3. Evaluation Metrics

Following standard semantic segmentation protocols, model performance was evaluated using Mean Intersection over Union (mIoU), Overall Accuracy (OA), and F1-score. The metrics are computed from the confusion matrix as follows:

(31) $${\mathrm{IoU}}_{i}=\frac{TP_{i}}{TP_{i}+FP_{i}+FN_{i}}$$

(32) $$\mathrm{mIoU}=\frac{1}{N}\sum_{i=1}^{N}{\mathrm{IoU}}_{i}$$

(33) $${F1}_{i}=\frac{2TP_{i}}{2TP_{i}+FP_{i}+FN_{i}}$$

(34) $$\mathrm{MeanF}1=\frac{1}{N}\sum_{i=1}^{N}{F1}_{i}$$

(35) $$\mathrm{OA}=\frac{\sum_{i=1}^{N}TP_{i}}{\sum_{i=1}^{N}\left(TP_{i}+FN_{i}\right)}$$

where *N* denotes the number of evaluated classes, and $TP_{i}$, $FP_{i}$, and $FN_{i}$ denote true positives, false positives, and false negatives for class *i*, respectively. Ignore labels and padded pixels are excluded from all metric calculations.

### 4.4. Comparative Analysis

#### 4.4.1. Performance on UAVid Dataset

**Figure 5:**
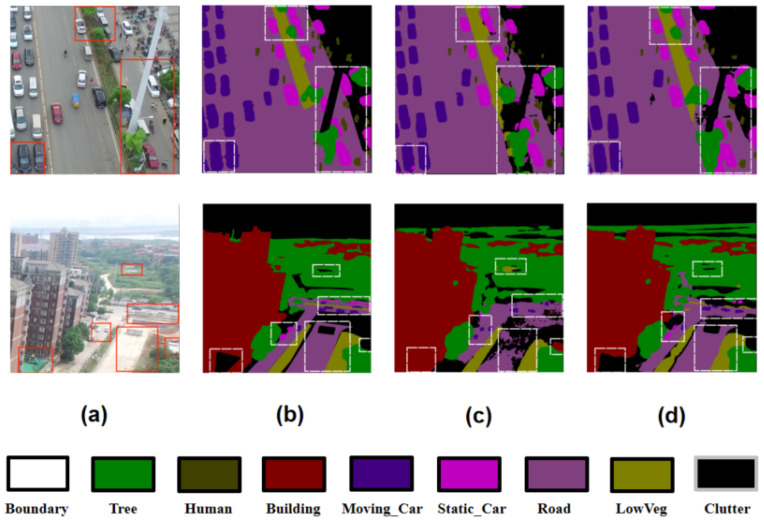
Qualitative comparison on the UAVid dataset. (**a**) Input Image, (**b**) Ground Truth, (**c**) UNetFormer, (**d**) DSD-Mamba. The proposed model is associated with clearer boundaries for small vehicles in cluttered scenes.

Table 1 presents the quantitative results on the UAVid dataset. DSD-Mamba achieves an mIoU of 73.4%, outperforming the Mamba-based baseline UMFormer [16] (67.6%) by 5.8 percentage points under the reported setting. In the small and dynamic categories, DSD-Mamba obtains per-class IoU scores of 76.7% for Moving Car and 50.0% for Human. The qualitative comparison in Figure 5 provides visual evidence for these numerical gains. In cluttered urban scenes, the baseline prediction tends to miss parts of small vehicles or merge them with surrounding background regions. In contrast, DSD-Mamba produces more compact and complete small-object regions, with fewer fragmented predictions around moving cars and pedestrians. This observation is consistent with the higher IoU values for Moving Car and Human in Table 1. These results suggest that the selective aggregation and global–local decoding design may help preserve small target responses in complex UAV scenes, although dedicated instance-level metrics would be required to further verify object completeness.

#### 4.4.2. Performance on ISPRS Vaihingen Dataset

**Figure 6:**
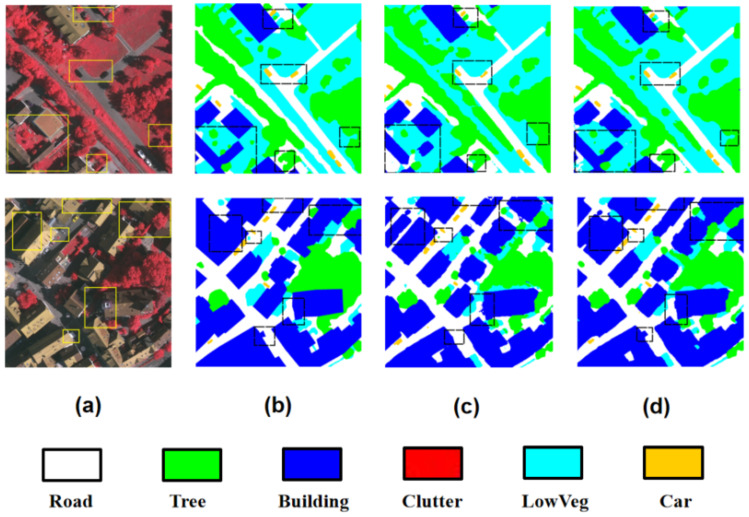
Qualitative comparison on the ISPRS Vaihingen dataset. (**a**) Input Image, (**b**) Ground Truth, (**c**) UMFormer, (**d**) DSD-Mamba. The proposed model shows clearer separation of clustered buildings and fewer isolated noisy predictions in this example.

Table 2 summarizes the comparative results on the multispectral ISPRS Vaihingen dataset. DSD-Mamba achieves an mIoU of 85.2% and an OA of 94.6%, which are the highest values among the listed methods under this protocol. It also obtains strong F1 scores for Impervious surfaces (97.3%) and Tree (91.7%), indicating favorable region-level performance in this evaluation. As illustrated in Figure 6, the prediction contains fewer isolated noisy regions and clearer separation of clustered buildings in the shown example.

For the ISPRS Vaihingen dataset, the visual comparison in Figure 6 highlights two typical urban parsing challenges: separating adjacent buildings and suppressing isolated noisy predictions. The baseline result shows local confusion around clustered building blocks, where neighboring roofs may be partially merged or boundary regions become less distinct. DSD-Mamba produces cleaner building regions and fewer isolated false predictions in the selected example. This visual observation is consistent with the quantitative results in Table 2, where DSD-Mamba achieves the highest mIoU and OA among the listed methods. The improvement may be associated with the combination of multi-scale feature aggregation and global–local decoding, but the boundary-related conclusion should remain qualitative because boundary F1 or trimap IoU is not reported.

#### 4.4.3. Performance on ISPRS Potsdam Dataset

**Figure 7:**
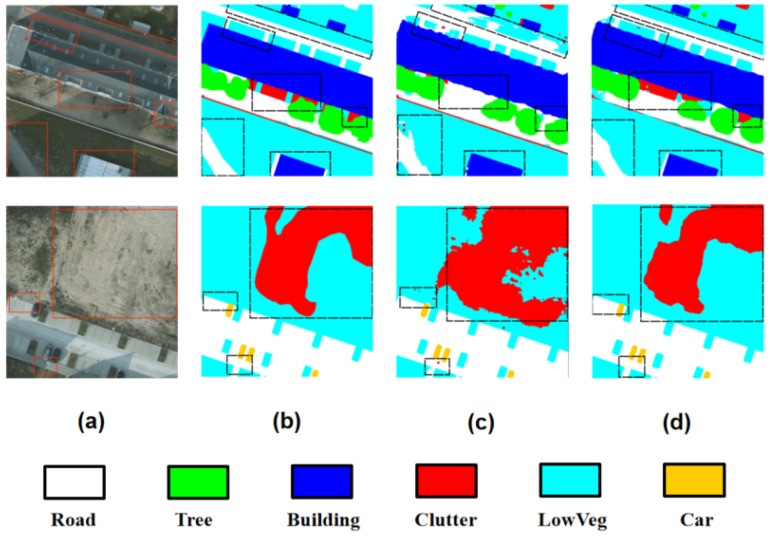
Qualitative comparison on the ISPRS Potsdam dataset. (**a**) Input Image, (**b**) Ground Truth, (**c**) UMFormer, (**d**) DSD-Mamba. The integration of SASA is associated with more continuous predictions for linear structures in this example.

For the high-resolution Potsdam dataset, Table 3 shows that DSD-Mamba achieves an mIoU of 87.2% under the same single-model inference protocol without TTA, which is 1.7 percentage points higher than UMFormer under the reported setting. The proposed model also obtains competitive F1 scores for Impervious surfaces, Buildings, Low vegetation, and Cars. The visual comparison in Figure 7 further clarifies where the improvement occurs. In the selected dense urban scene, the baseline prediction contains local discontinuities and fragmented regions along large surface areas and road-like structures. DSD-Mamba produces more spatially coherent impervious-surface and building regions, and the boundaries between adjacent land-cover categories are visually more stable. These observations are consistent with the strong F1 scores of Impervious surfaces, Buildings, and Cars in Table 3. The result suggests that SASA-guided axial context and DSCD-based global–local fusion may improve the spatial consistency of high-resolution predictions. However, because dedicated connectivity or boundary metrics are not included, this conclusion should be interpreted as qualitative support rather than direct topological evidence.

#### 4.4.4. Class-Wise Performance Analysis

Class-wise metrics are discussed as indirect evidence for small objects, elongated regions, and boundary-sensitive categories. These analyses are not intended to replace dedicated instance-level, boundary, or connectivity metrics.

Small-Object and Thin-Object Recognition: On the UAVid benchmark (Table 1), DSD-Mamba obtains IoU scores of 76.7% for Moving Car and 50.0% for Human, which are 5.3 and 19.7 percentage points higher than UMFormer [16], respectively. The observed gain is associated with the full model and may be related to the selective aggregation design in DSPFM; however, additional instance-level metrics would be needed to verify object completeness.

Elongated Structures: Impervious surfaces and Buildings are used as indirect indicators because dedicated connectivity metrics are not reported. On the ISPRS Potsdam dataset (Table 3), DSD-Mamba obtains F1 scores of 93.9% for Impervious surfaces and 97.5% for Buildings. Together with the qualitative examples, these results may be related to the axial context introduced by SASA, but they do not provide direct evidence of topological continuity.

Boundary-Sensitive Parsing: DSCD combines global semantic and local-detail streams during upsampling. The OA values of 94.6% on Vaihingen and 91.1% on Potsdam are consistent with stable pixel-level performance across the two urban datasets. Since Boundary F1 or trimap IoU is not reported, boundary-related observations remain qualitative and should be interpreted cautiously.

### 4.5. Ablation Study

An ablation study was conducted on the ISPRS Vaihingen dataset using a ResNet-18 U-shaped architecture without Mamba and attention modules as the baseline (Table 4). The baseline model yielded an mIoU of 83.3%. When evaluated individually, DSPFM, SASA, and DSCD obtained 84.0%, 84.4%, and 84.1% mIoU, respectively. These results indicate that each module provides a positive but moderate contribution under the adopted evaluation protocol.

The full model achieved the best result of 85.2% mIoU, corresponding to a 1.9 percentage-point improvement over the baseline. This improvement should be interpreted as moderate rather than dramatic. Since the baseline is already a strong U-shaped segmentation model and the Vaihingen benchmark is relatively mature, such an improvement may still be useful for high-resolution remote sensing segmentation. However, the gains from different modules were not strictly additive, suggesting that DSPFM, SASA, and DSCD interact with each other rather than contributing independently.

Therefore, the ablation results support the usefulness of integrating the three components, but they should not be interpreted as direct evidence that each module independently solves background suppression, elongated-structure continuity, or boundary refinement. In addition, mIoU is an overall class-averaged metric and may not fully capture improvements in boundary quality, small-object recovery, or linear-structure connectivity. More targeted metrics, such as boundary F1, trimap IoU, connectivity measures, and background-confusion analysis, would be needed to further isolate the exact role of each module.

To further examine the effect of the DSPFM alignment target resolution, we conducted an additional ablation study by comparing the default (1/32) alignment with an intermediate (1/16) alignment. In the (1/32) setting, all encoder features are aligned to the spatial resolution of ($E_{4}$). In the (1/16) setting, the features are aligned to the spatial resolution of ($E_{3}$), and the output is subsequently adapted to the decoder input scale to keep the remaining decoder structure unchanged. Therefore, the main experimental variable is the alignment resolution inside DSPFM.

As shown in Table 5, the intermediate 1/16 alignment does not improve the segmentation performance. Compared with the default 1/32 setting, the 1/16 setting decreases mIoU from 85.20% to 81.87% and OA from 94.61% to 93.81%. Although the 1/16 alignment preserves a denser spatial token grid, it also increases the affinity size by approximately 16× and may introduce more low-level texture redundancy and background heterogeneity into the bottleneck interaction stage. The class-wise results also show larger drops for Car and Clutter, suggesting that the higher-resolution interaction may make small or ambiguous categories more sensitive to noisy local responses. Therefore, the default 1/32 alignment is retained because it provides a better balance between compact semantic aggregation and segmentation accuracy under the adopted Vaihingen protocol.

### 4.6. Generalizability Across Datasets

To further discuss the generalizability of DSD-Mamba, we evaluated the model on three representative remote sensing semantic segmentation datasets: UAVid, ISPRS Vaihingen, and ISPRS Potsdam. These datasets exhibit clear differences in imaging platform, spatial resolution, scene composition, and annotation characteristics. UAVid contains high-resolution UAV video-frame imagery with complex urban scenes, strong perspective variations, and diverse object scales. In contrast, Vaihingen and Potsdam are aerial image benchmarks with nadir-view urban scenes, different ground sampling distances, and different distributions of buildings, vegetation, impervious surfaces, cars, and clutter.

The proposed model achieved mIoU scores of 73.4%, 85.2%, and 87.2% on UAVid, ISPRS Vaihingen, and ISPRS Potsdam, respectively. These results indicate that DSD-Mamba can maintain competitive segmentation performance across datasets with different spatial characteristics and scene distributions. This robustness can be attributed to the combination of three complementary designs: DSPFM performs compact multi-scale semantic aggregation at the bottleneck, SASA enhances directional context modeling for elongated structures, and DSCD combines global semantic modeling with local detail refinement during decoding.

It should be noted that these experiments evaluate dataset-level robustness under dataset-specific training and testing protocols. They do not represent strict zero-shot cross-dataset generalization, where a model trained on one dataset is directly tested on another dataset without fine-tuning. Such cross-dataset transfer is challenging because remote sensing datasets often differ in label definitions, annotation granularity, spatial resolution, imaging sensors, and scene distributions. We therefore consider cross-dataset transfer learning and domain generalization as important directions for future work.

### 4.7. Computational Complexity and Accuracy–Cost Trade-Off

To make the accuracy–cost relationship transparent, the computational complexity (FLOPs) and parameter count (Params) of DSD-Mamba are compared with representative models under the same 1024×1024 input size. As summarized in Table 6, DSD-Mamba requires 26.17 M parameters and 117.03 G FLOPs. Compared with the lightweight baseline UMFormer [16] (12.33 M parameters and 47.75 G FLOPs), DSD-Mamba remains more computationally expensive. The proposed method should therefore be viewed as accuracy-oriented rather than lightweight. It trades additional model capacity and spatial computation for higher segmentation accuracy under the reported Vaihingen protocol, while remaining smaller than Swin-UMamba in terms of parameters.

The additional cost mainly comes from modules that operate on spatial feature maps: the dense multi-scale pooling and selective aggregation in DSPFM, the strip-wise contextual modeling in SASA, and the multi-dilation local branch in DSCD. These operations increase FLOPs but are designed to retain multi-scale context and local high-frequency details that are relevant to small objects, thin structures, and object boundaries in very-high-resolution remote sensing imagery. Therefore, the term “sparse” in DSPFM should not be interpreted as a claim of reduced asymptotic complexity or reduced memory footprint. The measured FLOPs already include the dense affinity-logit computation and the additional spatial operations introduced by DSPFM, SASA, and DSCD. Under the adopted Vaihingen protocol, DSD-Mamba obtains 85.2% mIoU, which is 1.9 percentage points higher than UMFormer and 2.5 percentage points higher than UNetFormer. In terms of parameters, DSD-Mamba remains smaller than Swin-UMamba (26.17 M vs. 39.00 M), although it has higher FLOPs than the listed lightweight baselines. These results indicate an accuracy–cost trade-off that may be acceptable for offline mapping, post-disaster assessment, and other accuracy-sensitive remote sensing applications. For strictly real-time onboard deployment, further optimizations such as model compression, pruning, or knowledge distillation would still be needed.

### 4.8. Practical Applications and Deployment Considerations

The proposed DSD-Mamba framework has several potential practical applications in high-resolution remote sensing analysis. First, it can be used for urban land-cover mapping, where accurate pixel-level classification of buildings, impervious surfaces, vegetation, roads, and other land-cover categories is required for updating geographic information systems and supporting urban planning. Second, the improved modeling of elongated structures and local boundaries may be useful for extracting roads, bridges, rivers, and other linear or strip-like objects from aerial and UAV imagery. Such information can support transportation infrastructure analysis, municipal management, and map updating.

In addition, DSD-Mamba can be applied to UAV-based urban scene understanding. UAV imagery often contains complex object scales, occlusions, and perspective variations, and accurate semantic segmentation can assist traffic monitoring, vehicle-region analysis, and fine-grained urban inspection. For environmental and ecological applications, the segmentation of vegetation, impervious surfaces, and built-up areas can support green-space monitoring, land-cover change analysis, and urban environmental assessment. Furthermore, when appropriate task-specific training data are available, the framework may be adapted for post-event assessment tasks, such as identifying damaged built-up areas, blocked roads, or affected infrastructure after natural disasters.

From a deployment perspective, DSD-Mamba should be regarded as an accuracy-oriented model rather than a lightweight real-time model. Its relatively high FLOPs make it more suitable for offline processing, cloud-based mapping platforms, or workstation-level remote sensing production pipelines. Direct deployment on low-power edge devices may require additional compression or acceleration techniques. Therefore, future practical deployment will focus on model pruning, knowledge distillation, lightweight Mamba/CNN design, and runtime optimization.

## 5. Conclusions

In this paper, DSD-Mamba, an asymmetric dual-stream framework for high-resolution remote sensing semantic segmentation, was proposed. The architecture integrates CNN-based local representation, Mamba-based global context modeling, and Top-*k* selective feature aggregation. DSPFM, SASA, and DSCD are designed for selective multi-scale aggregation, axial skip-feature processing, and global–local decoding, respectively. Experiments on UAVid, ISPRS Vaihingen, and ISPRS Potsdam show that DSD-Mamba achieves mIoU scores of 73.4%, 85.2%, and 87.2%, respectively, under a single-model inference protocol without test-time augmentation. The consistent performance on these three datasets suggests that DSD-Mamba has reasonable dataset-level robustness under dataset-specific training protocols, although strict zero-shot cross-dataset generalization remains to be further investigated. The ablation results show that the full model is associated with higher mIoU than the baseline, but the module-level interpretations should remain cautious because dedicated background-suppression, connectivity, and boundary metrics are not reported. The complexity analysis shows that DSD-Mamba is an accuracy-oriented model rather than a lightweight one: it uses more FLOPs than lightweight baselines, while the added spatial computation is associated with higher mIoU in the reported experiments. Future work will focus on reducing computational cost through model compression, runtime optimization, and distillation. It will also investigate targeted boundary and connectivity metrics, multimodal extensions such as LiDAR–optical fusion, and cross-dataset transfer learning for stronger domain generalization. 

## Figures and Tables

**Table 1:** Quantitative comparison on the UAVid validation set. Per-class values denote IoU (%). The best results are highlighted in **bold**.

Method	Clutter	Bui.	Road	Tree	Veg.	Mo.Car	St.Car	Human	mIoU
SwiftNet [24]	64.1	85.3	61.5	78.3	**76.4**	51.1	62.1	15.7	61.1
MANet [25]	64.5	85.4	77.8	77.0	60.3	67.2	53.6	14.9	62.6
ABCNet [3]	67.4	86.4	81.2	**79.9**	63.1	69.8	48.4	13.9	63.8
Segmenter [9]	64.2	84.4	79.8	76.1	57.6	69.2	34.5	14.2	58.7
SegFormer [29]	66.6	86.3	80.1	79.6	62.3	72.5	52.5	28.5	66.0
BANet [30]	66.7	85.4	80.7	78.9	62.1	69.3	52.8	21.0	64.6
BoTNet [39]	64.5	84.9	78.6	77.4	60.5	65.8	51.9	22.4	63.2
CoaT [40]	**69.0**	88.5	80.0	79.3	62.0	70.0	59.1	18.9	65.9
DecoupleNet [41]	65.1	85.4	80.6	78.8	62.1	74.1	49.7	30.8	65.8
UNetFormer [4]	64.4	85.2	78.8	79.2	62.6	71.1	60.7	29.4	66.4
Mamba-UNet [13]	56.6	78.5	76.1	71.5	53.0	66.7	40.5	15.9	57.3
Swin-UMamba [14]	52.3	76.8	73.9	71.0	50.3	61.6	26.1	15.4	53.4
VM-UNet [12]	56.0	78.1	75.5	73.1	54.9	65.1	31.0	11.5	55.7
UrbanSSF-T [42]	65.0	85.7	79.1	79.6	63.5	69.0	54.7	29.3	65.7
UMFormer [16]	66.4	86.7	78.9	79.2	62.2	71.4	65.3	30.3	67.6
**DSD-Mamba (Ours)**	66.3	**91.5**	**81.3**	78.8	69.9	**76.7**	**73.0**	**50.0**	**73.4**

**Table 2:** Quantitative comparison on the ISPRS Vaihingen testing set. Per-class values denote F1 scores (%). The best results are highlighted in **bold**.

Method	Imp.surf.	Bui.	Lowveg.	Tree	Car	MeanF1	OA	mIoU
SwiftNet [24]	92.2	94.8	84.1	89.3	81.2	88.3	90.2	79.6
ABCNet [3]	92.7	95.2	84.5	89.7	85.3	89.5	90.7	81.3
FANet [43]	90.7	93.8	82.6	88.6	71.6	85.4	88.9	75.6
EaNet [26]	91.7	94.5	83.1	89.2	80.0	87.7	89.7	78.7
MAResU-Net [44]	92.0	95.0	83.7	89.3	78.3	87.7	90.1	78.6
BEDSN [45]	92.3	94.7	83.7	89.2	86.3	89.2	90.1	80.8
DPFE-AFF [46]	93.3	96.0	84.7	90.2	88.3	90.4	91.3	82.9
GANet [47]	93.1	95.9	84.6	90.1	88.4	90.4	91.3	–
TransUNet [8]	90.8	94.3	79.0	90.5	82.7	87.5	–	78.2
Swin-UperNet [28]	90.3	94.1	81.1	87.4	81.6	86.8	88.2	77.1
Segmenter [9]	89.8	93.0	81.2	88.9	67.6	84.1	88.1	73.6
BoTNet [39]	89.9	92.1	81.8	88.7	71.3	84.8	88.0	74.3
CMTFNet [31]	90.6	94.2	81.9	87.6	82.8	87.4	88.7	78.0
DCSA-Net [48]	92.1	96.2	83.0	90.3	82.4	88.8	90.6	78.9
ESDINet [49]	92.7	95.5	84.5	90.0	87.2	90.0	90.9	82.0
UNetFormer [4]	92.7	95.3	**84.9**	90.6	88.5	90.4	91.0	82.7
Mamba-UNet [13]	96.4	94.5	83.7	89.4	84.3	89.7	92.6	81.6
Swin-UMamba [14]	96.0	94.6	81.5	91.0	83.9	89.4	92.4	81.3
VM-UNet [12]	96.2	93.6	83.9	89.5	78.3	88.3	92.3	79.6
RS3Mamba [15]	92.8	**96.8**	80.8	91.1	**90.9**	90.5	–	82.8
UMFormer [16]	96.7	95.2	83.8	89.5	88.1	90.7	93.0	83.3
**DSD-Mamba (Ours)**	**97.3**	96.7	82.9	**91.7**	90.3	**91.8**	**94.6**	**85.2**

**Table 3:** Quantitative comparison on the ISPRS Potsdam testing set. Per-class values denote F1 scores (%). The reported DSD-Mamba result was obtained using single-model inference without test-time augmentation. The best results are highlighted in **bold**.

Method	Imp.surf.	Bui.	Lowveg.	Tree	Car	MeanF1	OA	mIoU
EaNet [26]	92.0	95.7	84.3	85.7	95.1	90.6	88.7	83.4
MAResU-Net [44]	91.4	95.6	85.8	86.6	93.3	90.5	89.0	83.9
SwiftNet [24]	91.8	95.9	85.7	86.8	94.5	91.0	89.3	83.8
FANet [43]	92.0	96.1	86.0	87.8	94.5	91.3	89.8	84.2
BEDSN [45]	91.8	95.6	85.9	86.7	95.0	91.0	89.2	83.8
DPFE-AFF [46]	92.3	95.4	85.8	87.5	93.7	90.5	89.7	82.8
ResUNet-a [27]	92.7	97.1	86.4	85.8	95.8	91.6	90.1	–
Swin-UperNet [28]	91.6	96.0	86.1	87.0	91.7	90.5	89.4	82.2
Segmenter [9]	91.5	95.3	85.4	85.0	88.5	89.2	88.7	80.7
CMTFNet [31]	92.1	96.4	86.4	87.3	92.4	90.9	89.9	83.6
ESDINet [49]	92.7	96.3	87.3	**88.1**	95.4	92.0	90.5	85.3
UNetFormer [4]	93.0	95.6	86.7	87.9	95.0	91.6	90.5	84.8
Mamba-UNet [13]	92.1	94.9	82.5	85.0	93.3	90.1	88.9	82.3
Swin-UMamba [14]	92.0	94.5	85.5	86.2	93.7	90.4	89.1	82.7
VM-UNet [12]	91.8	94.6	84.5	83.4	92.0	89.3	88.2	80.9
UMFormer [16]	93.7	96.4	86.7	87.8	95.5	92.0	90.9	85.5
**DSD-Mamba (Ours)**	**93.9**	**97.5**	**91.3**	84.9	**97.1**	**93.0**	**91.1**	**87.2**

**Table 4:** Ablation study on the ISPRS Vaihingen dataset. The best results are highlighted in **bold**. ✓ indicates that the corresponding module was included in the experiment, whereas “–” indicates that the module was not included.

DSPFM	SASA	DSCD	mIoU (%)	OA (%)
–	–	–	83.3	93.0
✓	–	–	84.0	94.2
–	✓	–	84.4	94.4
–	–	✓	84.1	94.1
✓	✓	–	84.5	94.3
✓	–	✓	84.7	94.4
–	✓	✓	84.9	94.5
✓	✓	✓	**85.2**	**94.6**

**Table 5:** Effect of DSPFM alignment resolution on the ISPRS Vaihingen dataset. The 1/32 setting corresponds to the default DSD-Mamba design. The relative affinity size is computed with respect to the 1/32 token grid. The best results are highlighted in **bold**.

Scale	Target	Grid	Rel. Affinity	mIoU (%)	OA (%)
1/32	$E_{4}$	32×32	1×	**85.20**	**94.61**
1/16	$E_{3}$	64×64	16×	81.87	93.81

**Table 6:** Comparison of computational complexity and parameters with an input size of 1024×1024. Lower Params/FLOPs indicate lighter models, while higher mIoU indicates better segmentation accuracy.

Method	Params (M)	FLOPs (G)	mIoU (%) on Vaihingen
UNetFormer [4]	11.84	54.70	82.7
Swin-UMamba [14]	39.00	96.00	81.3
UMFormer [16]	12.33	47.75	83.3
DSD-Mamba (Ours)	26.17	117.03	85.2

## Data Availability

The UAVid and ISPRS Vaihingen/Potsdam datasets are publicly available from the official UAVid website and the ISPRS 2D Semantic Labeling Contest websites [50,51,52]. The processed splits, training configurations, evaluation scripts, and code can be made available by the corresponding author upon reasonable request.
